# Degradation Efficiency and Mechanism of Tetracycline in Water by Activated Persulfate Using Biochar-Loaded Nano Zero-Valent Iron

**DOI:** 10.3390/molecules29163875

**Published:** 2024-08-15

**Authors:** Bojiao Yan, Xueqi Li, Xiaoyan Wang, Ping Yang, Hai Lu, Xiaoyu Zhang

**Affiliations:** 1 College of Visual Arts, Changchun Sci-Tech University, Changchun 130600, China; 100404@cstu.edu.cn; 2 School of Materials Science and Engineering, Changchun University of Science and Technology, Changchun 130022, China; lixueqi1027@163.com; 3 Key Laboratory of Songliao Aquatic Environment, Ministry of Education, Jilin Jianzhu University, Changchun 130118, China; wangxiaoyan@student.jlju.edu.cn (X.W.); haimm110@126.com (H.L.); 4 College of New Energy and Environment, Jilin University, Changchun 130021, China; yangping83@jlu.edu.cn

**Keywords:** biochar, nano zero-valent iron, persulfate, tetracycline, degradation efficiency, mechanism

## Abstract

Tetracycline (TC) contamination in water is one of the key issues in global environmental protection, and traditional water treatment methods are difficult to remove antibiotic pollutants.Therefore, efficient and environmentally friendly treatment technologies are urgently needed. In this study, activated persulfate (PS) using a biochar-loaded nano zero-valent iron (BC-nZVI) advanced oxidation system was used to investigate the degradation effect, influencing factors, and mechanism of TC. BC-nZVI was prepared using the liquid-phase reduction method, and its structure and properties were analyzed by various characterization means. The results showed that nZVI was uniformly distributed on the surface or in the pores of BC, forming a stable complex. Degradation experiments showed that the BC-nZVI/PS system could degrade TC up to 99.57% under optimal conditions. The experiments under different conditions revealed that the iron-carbon ratio, dosing amount, PS concentration, and pH value all affected the degradation efficiency. Free radical burst and electron paramagnetic resonance (EPR) experiments confirmed the dominant roles of hydroxyl and sulfate radicals in the degradation process, and LC–MS experiments revealed the multi-step reaction process of TC degradation. This study provides a scientific basis for the efficient treatment of TC pollution in water.

## 1. Introduction

With the advancement of medical care and the development of animal husbandry, antibiotics, as antimicrobial drugs that kill or inhibit the growth of bacteria, are widely used in the treatment of microbial infections in both humans and livestock [1,2]. Klein et al. [3] found that global antibiotic consumption, expressed in terms of the defined daily doses (DDDs), increased by 65% between 2000 and 2015 (21.1–34.8 billion doses), and the rate of antibiotic consumption increased by 39% (11.3–15.7 doses per 1000 people per day). During this period, antibiotic consumption in China surged by 79% (2.3–4.2 billion doses). According to incomplete statistics, the annual production of antibiotics in China is about 210,000 tons, which has become the largest antibiotic producer and consumer in the world [4,5]. This shows that the use of antibiotics in China, and even worldwide, is very large and increasing [6]. According to the differences in their chemical structures, antibiotics can be divided into several categories, including β-lactams, macrolides (MAs), fluoroquinolones (FQs), tetracyclines (TCs), and sulfonamides (SAs) [7,8]. Among them, TCs inhibit microbial protein synthesis by binding to the 30S subunit of the microbial ribosome and thus inhibit bacterial growth [9,10]. TCs exhibit activity against many atypical microorganisms such as Gram-positive and Gram-negative bacteria, protozoan parasites, mycoplasma, Chlamydia, and Rickettsia. It has become one of the most commonly used broad-spectrum antibiotics for the treatment of bacterial infections in humans and animals due to its antimicrobial activity and minimal side effects [11].

Studies have shown that organisms have a limited ability to metabolize antibiotics, with only a small amount being absorbed into the body and most of it being excreted into the environment in the form of parent compounds through urine and feces [12]. The excessive use and unregulated discharge of antibiotics have resulted in large amounts of residual antibiotics in water bodies, and conventional wastewater treatment systems are inadequate in removing these residual antibiotics, which has led to their prevalence in soils, surface water, groundwater, and even drinking water [13,14,15]. TCs belong to the category of refractory organic pollutants, which are highly stable, soluble, and biotoxic and can have a negative impact on the structure and function of the microbial communities in the water body [16]. In addition, TCs can lead to the development and spread of bacterial resistance and affect the clinical effectiveness of antibiotics [17,18]. Thus, while antibiotics bring convenience to human beings, they also pose a serious threat to aquatic organisms and human health. Therefore, the treatment of TCs is urgent.

Traditional physicochemical methods such as adsorption, coagulation and precipitation, and membrane separation are difficult to effectively remove TC from water. These methods can only transfer the pollutants to other locations, fail to achieve their complete degradation, and may generate a large number of secondary pollutants [12,19]. Therefore, there is a need to develop new, efficient, low-cost, and environmentally friendly technologies to treat this difficult-to-degrade pollutant. In recent years, advanced oxidation technologies (AOPs) based on free radical oxidation reactions have attracted much attention. This technology utilizes strongly oxidizing free radicals, such as hydroxyl radical (·OH) and sulfate radical (SO_4_^−·^), to attack the chemical bonds of organic pollutants in water in order to mineralize or render them harmless [20,21,22]. Persulfate (PS) is a commonly used radical precursor that can decompose to form SO_4_^−·^ and ·OH under certain conditions with high oxidizing capacity and stability [23,24]. However, the decomposition rate of PS itself is slow and certain activators need to be added to improve its reaction efficiency [25].

Biochar (BC) is a carbonaceous material produced by low-temperature pyrolysis of waste biomass, which has the advantages of abundant functional groups, low cost, and renewability [26,27,28]. Nano zero-valent iron (nZVI) is a metallic nanomaterial with high reducing and catalytic properties, which can undergo an electron transfer reaction with PS and promote its decomposition to generate free radicals [29]. Loading nZVI onto BC can overcome the drawbacks of nZVI such as easy aggregation, oxidation, and deactivation, and improve its dispersion, stability, and utilization, as well as enhance the enrichment and contact with TC in water by utilizing the adsorption of BC, thus improving the degradation efficiency [30,31]. Therefore, this study aimed to investigate the mechanism, influencing factors and kinetic model of TC degradation in water by BC-nZVI-activated PS, to provide theoretical basis and technical support for the engineering application of this technology, and also to provide theoretical basis and practical experience for the large-scale treatment and disposal of antibiotic wastewater, which is of far-reaching significance.

## 2. Results and Discussion

### 2.1. Analytical Testing and Micro-Characterization Methods

#### 2.1.1. Analytical Test Methods

(1) Detection of TC concentration

**Figure 1:**
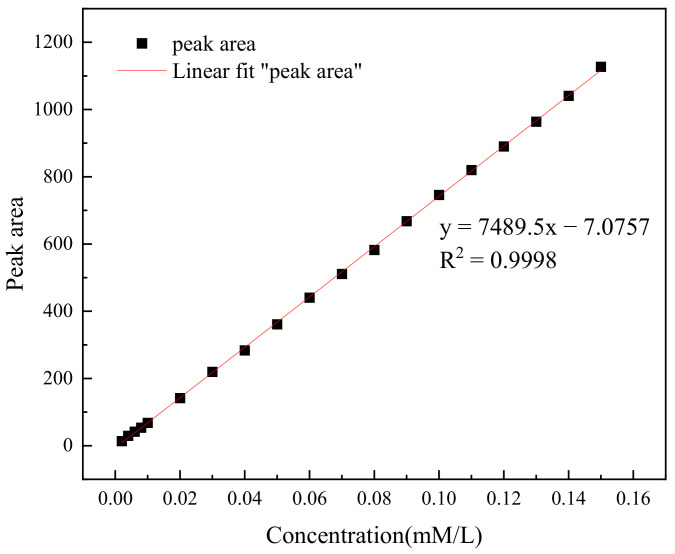
TC standard curve.

Liquid chromatography (HP–LC) was used to detect the concentration of TC in water. A series of solutions of TC with a concentration gradient of 0.002–0.15 mM were configured, respectively, and the concentration of TC was determined by HPLC, and the standard curve of TC was obtained as in Figure 1. The TC content was analyzed by an Agilent 1260 liquid chromatograph using an SBC-18 column (5 mm × 150 mm × 4.6 µm). The liquid chromatographic conditions were as follows: column temperature 35 °C, column flow rate 1 mL/min.

(2) Electron paramagnetic resonance (EPR)

In this experiment, EPR was used to measure the type and amount of radicals in the oxidation reaction under different conditions. A special spin trapping agent, 5,5-dimethyl-1-pyrroline-N-oxide (DMPO), was used in the experiment, which can form stable adducts with free radicals, resulting in characteristic EPR signals. By analyzing the EPR spectra of DMPO adducts, the type and relative content of free radicals can be determined.

(3) Liquid chromatography–mass spectrometry (LC–MS) analysis

The degradation products of TC were derived by analyzing using a liquid mass spectrometer of Thermo Fisher Scientific brand model Thermo Scientific TSQ Fortis Plus, USA. The test conditions were as follows: the inlet temperature was 290 °C, the carrier gas was high-purity helium at a flow rate of 20 mL·min^−1^, and the split ratio was non-split sampling. The heating procedure of LC–MS was as follows: the initial temperature was 80 °C with a retention time of 1 min; the temperature was then increased at a rate of 3 °C·min^−1^ to 161 °C, and then to 220 °C at 5 °C·min^−1^; and the temperature was finally increased at a rate of 10 °C·min^−1^ to 300 °C with a retention time of 5 min. Finally, the running was continued at 300 °C for 5 min.

(4) Free radical quenching method

The free radical quenching method is a method that utilizes a free radical scavenger to react with free radicals, thereby inhibiting or eliminating the free radical reaction. In this experiment, four different free radical scavengers were selected, namely tert-butanol (TBA), methanol (MeOH), histidine (His), and p-benzoquinone (PBQ). TBA has a high reaction rate constant with ·OH and can effectively scavenge ·OH radicals. MeOH has a high reaction rate constant with both ·OH and SO_4_^−·^ and can scavenge both radicals. PBQ has a high reaction rate constant with ·O_2_^−^ and can effectively scavenge the ·O_2_^−^ radical. His can be used as a scavenger for ^1^O_2_. By comparing the reaction results after adding different scavengers, it is possible to determine which radicals are present in the reaction.

#### 2.1.2. Micro-Characterization Methods

(1) X-ray photoelectron spectroscopy (XPS)

After taking an appropriate amount of samples pressed and attached to the sample disk, the samples were put into the sample chamber of the Thermo Scientific K-Alpha XPS instrument. The samples were fed into the analysis chamber when the pressure in the sample chamber was less than 2.0 × 10^−7^ mbar, with a spot size of 400 μm, an operating voltage of 12 kV, and a filament current of 6 mA; the full-spectrum scanning fluence energy of 150 eV and a step size of The full-spectrum scanning fluence energy was 150 eV with a step size of 1 eV; the narrow-spectrum scanning fluence energy was 50 eV with a step size of 0.1 eV.

(2) X-ray diffraction (XRD)

X-ray diffraction experiments were carried out using a Japanese Rigaku SmartLab SE instrument with a copper target. The scanning range was set to 5–90 degrees and the scanning rate was 5 degrees per minute. Jade 9.0 software was used for data processing and analysis.

(3) Fourier transform infrared spectroscopy (FT-IR)

In a dry environment, a sample visible to the naked eye and an appropriate amount of dry potassium bromide powder were added to a mortar and pestle, ground thoroughly for several times. Then put it into a tablet press to press the tablets, and the test was performed by acquiring the background first, and then acquiring the infrared spectra of the sample with a resolution of 4 cm^−1^, the number of scans was 32, and the range of test wave numbers was 400/600–4000 cm^−1^.

(4) Scanning electron microscope-energy scattering spectroscopy (SEM-EDS)

Take a trace amount of sample directly stick to the conductive adhesive and use Quorum SC7620 shot plating instrument spray gold 45 s, spray gold for 10 mA; then use TESCAN MIRA LMS scanning electron microscope to shoot the sample morphology, energy spectrum mapping and other tests. The accelerating voltage when the morphology shooting is 3 kV, and the accelerating voltage when the energy spectrum mapping shooting is 15 kV. The detector for the SE2 secondary electron detector.

(5) Specific surface area and porosity analyzer (BET)

The sample was pretreated for 8 h using the standard degassing station of Mike’s instrument under the condition of vacuum 200 °C. Then, under the condition of 77 k liquid nitrogen, the nitrogen adsorption and desorption test of the sample was carried out by using a 4-station automatic specific surface area analyzer of American Micromeritics APSP 2460 model. Then the isothermal suction–desorption curves were obtained when the instrument analysis was completed, and the total specific surface area of the materials was obtained through the BET method.

### 2.2. Material Properties and Functional Characterization of Activated Systems

#### 2.2.1. Characterization and Analysis of Microscopic Morphology and Characteristic Elements

**Figure 2:**
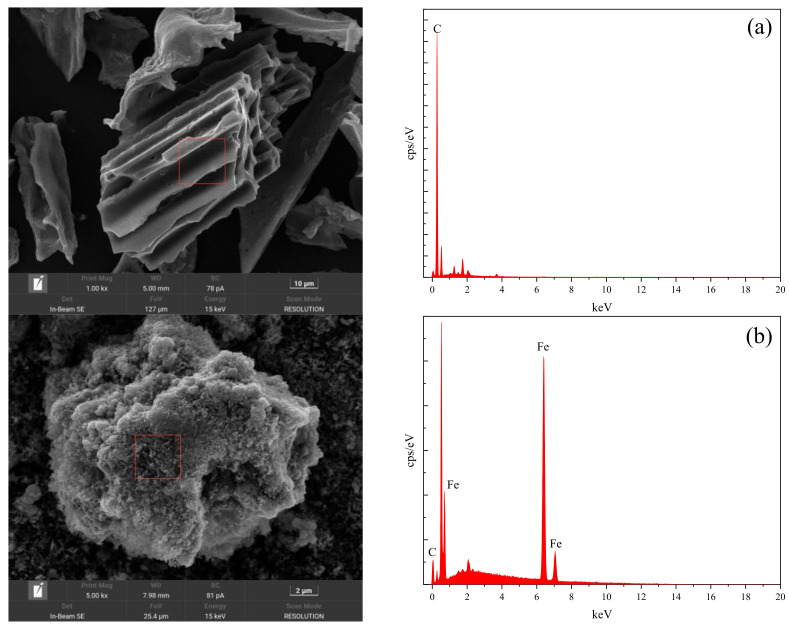

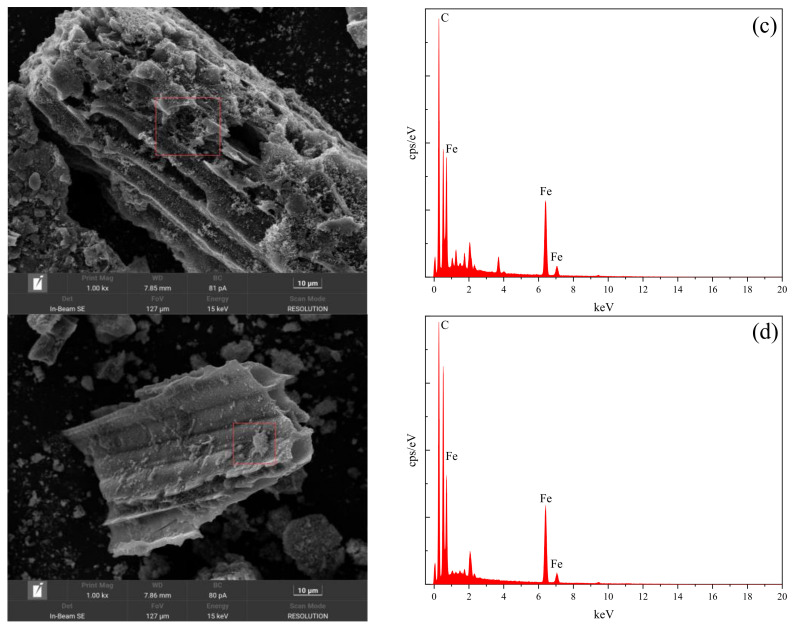
SEM-EDS diagram of BC (**a**), nZVI (**b**), BC-nZVI (new) (**c**), and BC-nZVI (used) (**d**).

The use of SEM-EDS to characterize and analyze the microscopic morphology and characteristic elements of a material is a commonly used material characterization method, which can provide information on the surface morphology, elemental distribution, and chemical composition of a material. The principle of SEM-EDS is to irradiate the surface of a material with a high-energy electron beam generated by a scanning electron microscope (SEM), which induces the material to emit different types of secondary electrons and X-rays, and these signals can be detected to obtain a morphological image and an elemental spectrum of the material. By detecting these signals, the topographic image and elemental spectrum of the material can be obtained. EDS can use the relationship between the energy of the X-rays and the emitted elements to determine which elements are present in the material and the relative content of each element. SEM-EDS can analyze different areas or points quantitatively or semi-quantitatively, so as to reveal the microstructural and compositional characteristics of the material. In this experiment, SEM-EDS was used to observe and analyze four materials, BC, nZVI, BC-nZVI (new), and BC-nZVI (used), and the characterization results are shown in Figure 2.

From Figure 2a, it could be seen that BC was mainly composed of carbon, oxygen, silicon, and calcium, and its surface had a porous mesh structure, which was favorable for the loading of nZVI and the adsorption of heavy metal ions. Figure 2b showed that nZVI was mainly composed of iron, oxygen, carbon, and other elements, and it showed an irregular spherical or lamellar structure with high specific surface area and reactivity but was easy to agglomerate. Figure 2c showed that BC-nZVI (new) was a compact composite formed by nZVI particles uniformly distributed on the surface or in the pores of BC. From Figure 2d, it could be seen that the surface morphology of the activated material (BC-nZVI (used)) after the reaction was significantly changed, with many small holes and cracks, while the iron content was significantly reduced, but the EDS spectrum still showed the presence of an Fe element. This meant that the reacted nZVI was not completely oxidized and retained a certain reducing capacity, which could be continued to use for degrading other pollutants with good reusability.

#### 2.2.2. Characterization and Analysis of Crystal Structure and Phase Composition

**Figure 3:**
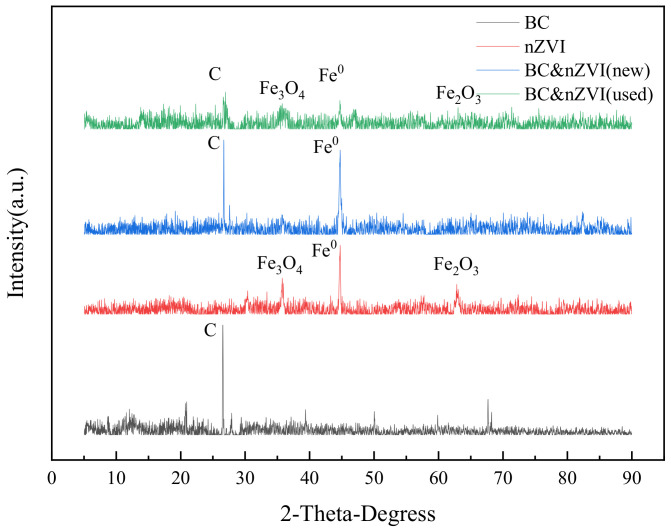
XRD results of BC, nZVI, BC-nZVI (new), and BC-nZVI (used).

An X-ray diffractometer (XRD) is an instrument that utilizes the diffraction interaction between X-rays and crystals to study the structure of matter. It can qualitatively analyze samples with different physical phases to determine their crystal structure, cell parameters, crystal plane spacing, grain size, stress state and other information. XRD is a non-destructive analytical method suitable for solid materials. In this experiment, the XRD results of the samples were checked against the standard PDF cards of the physical phases to complete the qualitative analysis of the physical phase composition and structure of the materials. The XRD results of BC, nZVI, BC-nZVI (new), and BC-nZVI (used) XRD characterization results are shown in Figure 3.

BC is a carbon-based material made from biomass by pyrolysis or sintering, which is mainly composed of carbon and oxygen and has an amorphous structure. As can be seen in Figure 3, the diffraction peak of BC appears at 2θ = 27.2° [32]. The nZVI is pure metallic iron in cubic crystal system with strong diffraction peaks. The nZVI’s XRD characterization shows that the absorption peak at 2θ = 44.7° is the apparent peak of Fe^0^, and its peak position and intensity are consistent with that of the standard card, while the peaks at 2θ = 36.5° and 2θ = 62.7°. The peaks at 2θ = 36.5° and 2θ = 62.7° represent Fe_3_O_4_ and Fe_2_O_3_, respectively, which indicates that part of nZVI was still oxidized during the preparation of nZVI, although it was protected by N_2_ atmosphere [33]. As shown in Figure 3, BC-nZVI (new) had the characteristic peaks of BC and nZVI at the same time, which indicates that the loading of nZVI on BC was successful and that no chemical reaction occurred between nZVI and BC, and only physical mixing occurred. At the same time, no obvious heterogeneous peaks appeared in the figure, which proved that the loading could effectively inhibit nZVI from being oxidized.In the XRD characterization results of BC-nZVI (used), in addition to the characteristic peaks of BC and nZVI, the diffraction peaks of iron oxides, such as Fe_3_O_4_, Fe_2_O_3_, etc., appeared, indicate that nZVI was oxidized during the reaction process and different oxides were generated.

#### 2.2.3. Characterization and Analysis of Specific Surface Area and Pore Properties

**Figure 4:**
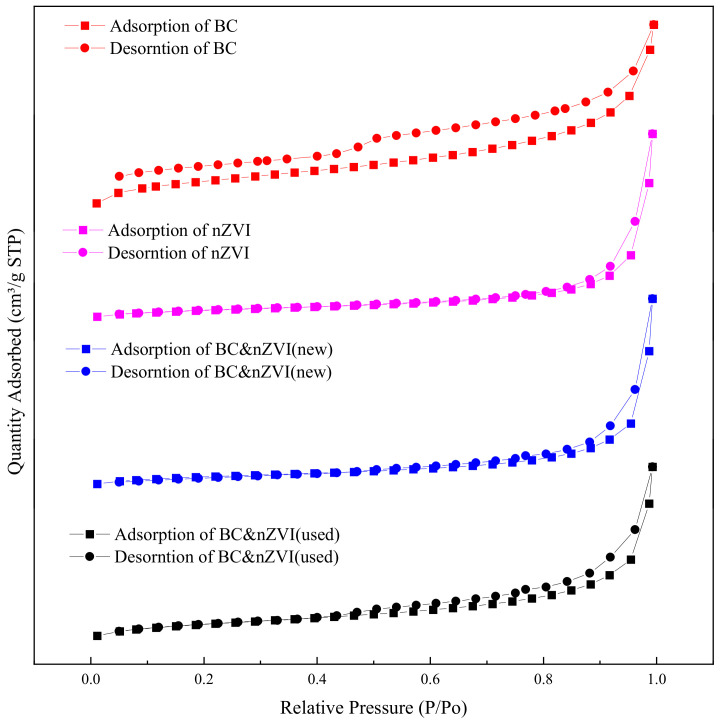
N_2_ adsorption–desorption isotherms of different materials.

BET analysis is a commonly used method for evaluating nanomaterials, which is a model for calculating the number of molecules of a gas absorbed on the surface of a solid. The BET specific surface area is the ratio of the surface area occupied by a gas molecule adsorbed to the surface of a solid to the mass of the solid when the gas molecule is adsorbed to the surface of the solid to form a monolayer at a certain temperature and pressure. Pore characterization refers to the measurement of parameters such as the size, distribution, and number of pores in a solid material by gas adsorption, and the pore characteristics play an important role in influencing the catalytic, adsorption, transport, and other properties of the material. Four materials, BC, nZVI, BC-nZVI (new), and BC-nZVI (used), were tested by BET, and the obtained N_2_ adsorption–desorption isotherms were shown in Figure 4, and the parameters of BET specific surface area, average pore size, and total pore volume of different materials were shown in Table 1.

From Table 1, it could be seen that the specific surface areas of BC and nZVI were 36.419 m^2^/g and 43.928 m^2^/g, respectively, which indicated that these two materials had high specific surface areas, which were favorable for the adsorption of pollutants in water. The specific surface area of BC-nZVI (new) was 32.9537 m^2^/g, which was lower than that of BC and nZVI, probably because nZVI was partially filled with BC during the preparation process. The specific surface area of BC-nZVI (used) was 91.3207 m^2^/g, which was much higher than that of the other three materials, which may be due to the fact that nZVI underwent a redox reaction during the reaction process, which produced new oxides and increased the specific surface area of the material [34]. From Figure 4, it could be seen that the isotherms of all four materials showed obvious hysteresis curves, indicating that these materials had certain pore structures. Based on the shape of the hysteresis curves, it could be judged that all four materials belong to type IV isotherms, indicating that these materials were mainly composed of mesopores [35]. This was corroborated by the data of average pore size and total pore volume.

#### 2.2.4. Chemical Composition and Functional Group Characterization and Analysis

**Figure 5:**
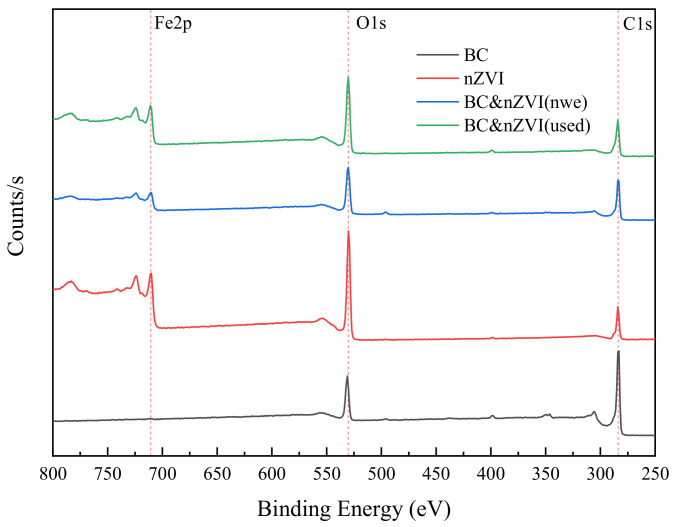
XPS diagram of different materials.

XPS analysis of chemical composition is a surface analysis technique that can measure the type, content and chemical state of elements on the surface of a material. XPS uses X-rays to excite photoelectrons on the surface of a material, and then analyzes the energy and intensity of the photoelectrons through an energy spectrometer to obtain chemical information on the surface of the material. In order to investigate the chemical composition of the materials, XPS was used in this experiment to analyze the powdered BC, nZVI, BC-nZVI (new), and BC-nZVI (used) materials, and the results were shown in Figure 5.

From Figure 5, it could be seen that the BC samples were mainly composed of elements C and O. The binding energies of C1s and O1s were 284.8 eV and 532.5 eV, respectively, indicating that a large number of oxygen-containing functional groups, such as carboxyl, hydroxyl, and ester groups, existed in BC [36]. nZVI samples, in addition to the elements C and O, also contained the element Fe, and the binding energy of Fe_2_p was 707.6 eV, which indicated that nZVI existed mainly in the form of Fe^0^. The elemental composition of the BC-nZVI (new) sample was similar to that of the nZVI sample, but the binding energy of Fe_2_p was slightly lower at 706.9 eV, indicating that a certain degree of interaction between nZVI and BC had occurred [37]. Compared to the BC-nZVI (new), the BC-nZVI (used) sample’s elemental composition changed significantly, with the presence of elements such as N, S, and Cl, in addition to C, O, and Fe. This might originate from pollutants such as nitrogen, sulfur, and chloride in the water. Meanwhile, the binding energy of Fe_2_p was elevated to 709.4 eV, indicating that nZVI was oxidized to Fe^2+^ or Fe^3+^ in the redox reaction.

**Figure 6:**
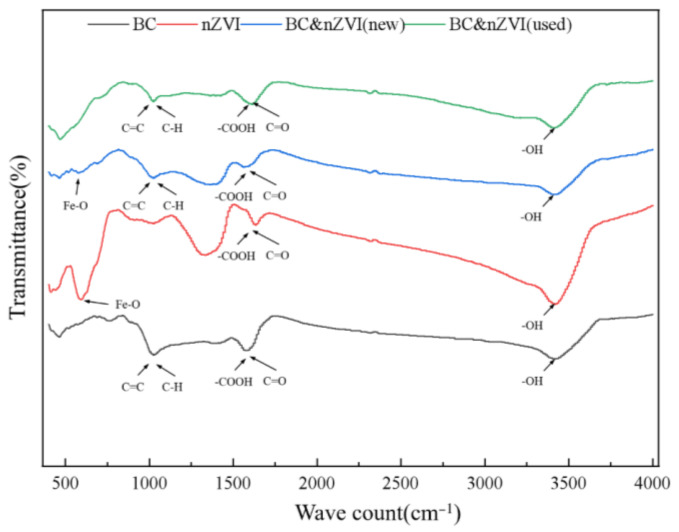
FT-IR diagram of different materials.

FT-IR is a method of determining the chemical structure and composition of a substance by measuring its infrared absorption or transmission properties using an infrared spectrometer. The principle of FT-IR functional group characterization and analysis is based on the fact that atomic bonds in a molecule selectively absorb or radiate energy to infrared light at a specific frequency to form an infrared spectrum. The absorption peaks or transmission peaks in the infrared spectrum can reflect the different types of functional groups present in the molecule, such as carbonyl, hydroxyl, amino, halogen, etc. In this experiment, FT-IR was used to analyze the powdered BC, nZVI, BC-nZVI (new), and BC-nZVI (used) materials, and the results are shown in Figure 6.

From Figure 6, it could be seen that there are some characteristic peaks in the spectra of BC, such as O–H stretching vibration peak at 3420 cm^−1^, C–H stretching vibration peak at 2920 cm^−1^, C=O stretching vibration peak at 1630 cm^−1^, C–O stretching vibration peak at 1420 cm^−1^, and C–O–C stretching vibration peak at 1050 cm^−1^, which indicated that BC contained functional groups such as hydroxyl, carbonyl, alcohol, and phenol groups [38]. The nZVI had only one weak peak located at 460 cm^−1^ in its spectrum, which might be due to the presence of Fe–O bonds [39]. The BC-nZVI (new) had a stronger peak located at 570 cm^−1^ in its spectrum in addition to retaining the characteristic peaks of BC, suggesting that nZVI had successfully loaded on the BC. Meanwhile, the Fe–O–Cbonds formed. The O–H peak at 3420 cm^−1^ and the C=O peak at 1630 cm^−1^ were significantly weaker in the spectrum of BC-nZVI (used) compared with that of BC-nZVI (new), suggesting that the hydroxyl and carbonyl groups on the surface of the biochar were depleted during the degradation process, which might be due to the reaction with the pollutants. Meanwhile, the Fe−O–C peak at 570 cm^−1^ was also weakened, indicating that nZVI was oxidized during the degradation process.

### 2.3. Laws and Control Factors of TC Degradation in Water by BC-nZVI/PS System

#### 2.3.1. Characterization of the Degradation of TC in Water by Reaction Systems of Different Classes of Activated Materials

**Figure 7:**
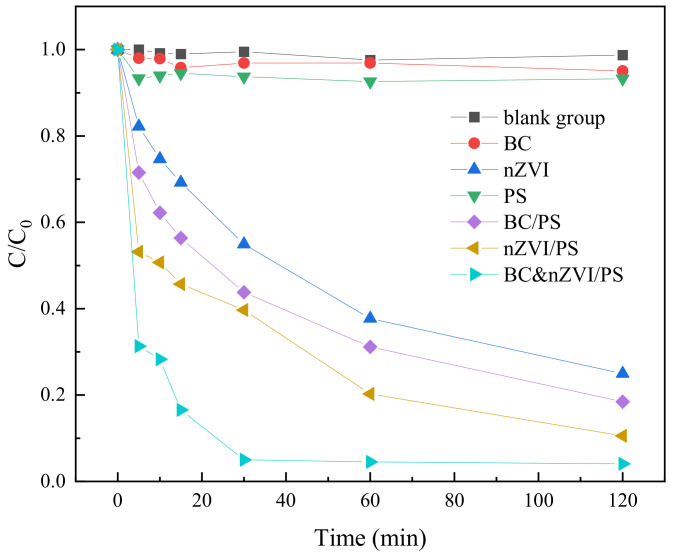
The degradation results of TC under different systems.

In order to investigate the activation of persulfate (PS) by biochar-loaded nano zero-valent iron (BC-nZVI) and its degradation efficiency of TC, six different oxidation reaction systems were designed, namely, PS, BC, BC/PS, nZVI, nZVI/PS, and BC-nZVI/PS, respectively. Reaction conditions of each system are shown in Table 2. During the experiments, 10 mg of PS activation material was added to 100 mL of TC solution at an initial concentration of 0.05 mM for each system, and the dosage of PS was 0.25 mM. The solutions were then placed in a thermostatic oscillator and stirred homogeneously at a speed of 200 r/min. At different time points, the solution samples were taken out and filtered through 0.45 μm filter membrane, and then the concentration of TC was determined by HPLC as a way to evaluate the degradation efficiency of each system on TC. The degradation results are shown in Figure 7.

Figure 7 shows the degradation results of TC under PS, BC, BC/PS, nZVI, nZVI/PS, and BC-nZVI/PS systems, respectively. First, the degradation rates of TC using BC, nZVI or PS alone, and the blank control group were compared. The results showed that the degradation effect of BC or PS alone was poor, with degradation rates of 5.07% and 6.95% within 120 min, respectively, while the degradation effect of nZVI alone was better, with a degradation rate of 77.72% within 120 min. This indicated that nZVI could directly react with TC in an electron transfer reaction, thus inactivating TC. The lower activity of BC and PS might be attributed to the fact that BC has fewer surface defects and cannot effectively adsorb and activate PS, while PS has a slower self-decomposition rate and cannot produce enough SO_4_^−·^ to oxidize TC. Secondly, the degradation rates of the three combined reaction systems, namely, BC/PS, nZVI/PS, and BC-nZVI/PS, were compared with respect to TC. The results showed that the degradation effects of the three combined reaction systems were better than those of BC, nZVI or PS alone, with BC-nZVI/PS showing the best degradation effect, with a degradation rate of 99.34% in 120 min. This indicated that the synergistic effect of BC, nZVI and PS could significantly improve the degradation efficiency of TC. Specifically, BC could provide a large amount of surface area and pore structure, thus increasing the loading and contact area of nZVI and PS, while the surface functional groups of BC could absorb and activate PS to promote the production of SO_4_^−^·. Then nZVI could undergo a Fenton reaction with PS to produce OH·, which increased the type and concentration of oxidant [40]. At the same time, nZVI could also undergo direct electron transfer reaction with TC to accelerate the degradation of TC. While PS could be activated by BC and nZVI to produce SO_4_^−^· and OH·, which oxidatively degraded TC [41]. Therefore, it can be concluded that the BC-nZVI/PS system had the best TC removal efficiency compared with other oxidation systems, and the BC-nZVI material could effectively catalyze the activation of PS to generate enough active radicals to degrade TC in water.

#### 2.3.2. Effect of Different Iron–Carbon Ratios on the Degradation of TC in Water

**Figure 8:**
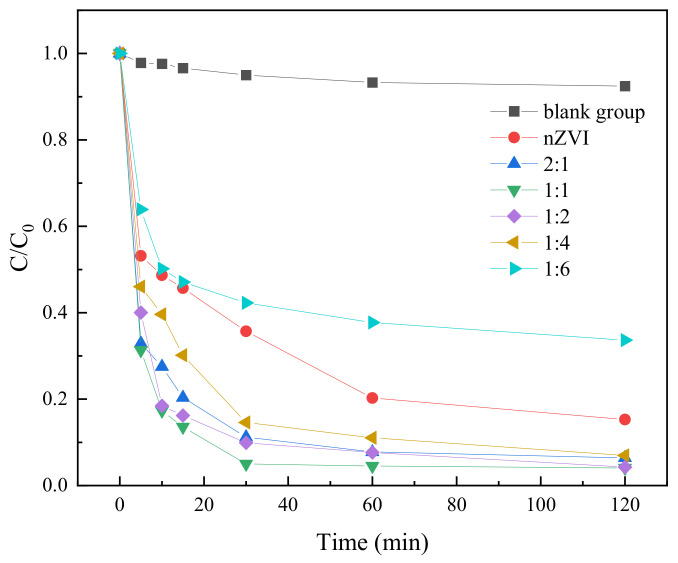
The degradation results of TC under different iron-carbon ratios.

In order to investigate the effect of different iron-carbon ratios in the activation materials on the degradation of TC, five activation materials with different iron-carbon ratios, namely, 2:1, 1:1, 1:2, 1:4, and 1:6, were designed. During this experiment, 10 mg of these five materials with different iron-carbon ratios were added into 100 mL of TC solution with an initial concentration of 0.05 mM. The amount of PS dosed was 0.25 mM. Then the solutions were placed in a constant temperature oscillator and stirred uniformly at a speed of 200 r/min. Then at different time points, the solution samples were taken out and filtered through a 0.45 μm filter membrane, and then the concentration of TC was determined by HPLC to evaluate the degradation efficiency of the activated materials with different iron-carbon ratios on TC. The results were shown in Figure 8.

In Figure 8, it was shown that the degradation of TC by materials with different iron to carbon ratios had significant differences, among which the activated material with an iron–carbon ratio of 1:1 exhibited the highest degradation efficiency of 99.34%. This might be because that this activated material had a higher iron content, which provided more active iron sites and promoted the decomposition of PS and the generation of ·OH. In addition, the activated material also had a certain carbon content, which could absorb TC and increase its contact opportunity with ·OH to improve its degradation rate. The degradation effect of the activated material with iron–carbon ratios of 1:2, 2:1, 1:4, and 1:6 on TC gradually decreased to 99.11%, 96.35%, 92.14%, and 68.71%, respectively. This might be attributed to the fact that with the increase of carbon content, the iron content decreases accordingly, resulting in insufficient iron sites, and the decomposition of PS and the generation of ·OH were limited. Although the increase of carbon content could enhance the adsorption capacity of TC, the degradation rate of TC was inhibited due to the insufficiency of ·OH [42]. In addition, too high carbon content may also lead to structural instability of the activated materials and reduced iron dispersion and activity.

#### 2.3.3. Effect of BC-nZVI Dosage on TC Degradation

**Figure 9:**
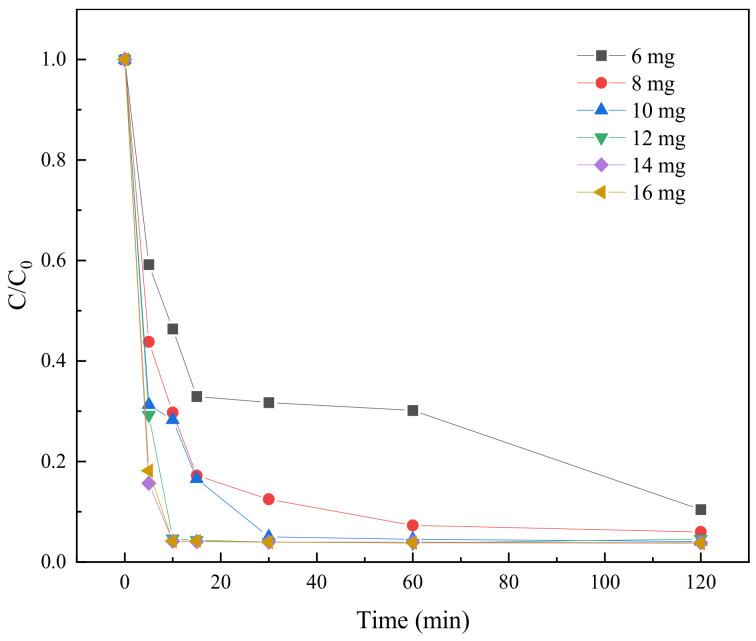
The degradation results of TC under different BC-nZVI dosages.

In order to investigate the effect of BC-nZVI dosage on TC degradation, six different dosages of activation materials with iron to carbon ratios of 1:1, namely 6 mg, 8 mg, 10 mg, 12 mg, 14 mg, and 16 mg, were selected. In the experimental process, six different dosages of activation materials were added into 100 mL of TC solution with an initial concentration of 0.05 mM, and the PS The dosage of PS was 0.25 mM. The TC degradation effects with different dosages of activation materials are shown in Figure 9.

It can be seen from Figure 9 that the degradation rate of TC increased rapidly in a short period of time with the increase of material dosage, especially in the range of 10 mg to 16 mg. For example, at 5 min, the dosage of 6 mg achieved only a 42.23% degradation rate, while the dosage of 16 mg achieved 84.73%. As the reaction time increased, the degradation rate increased for all dosages, but the magnitude of the increase varied. At 120 min, the dosage of 6 mg achieved a 92.77% degradation rate while the dosage of 16 mg reached 99.70%.

#### 2.3.4. Effect of Initial TC Concentration on Degradation

**Figure 10:**
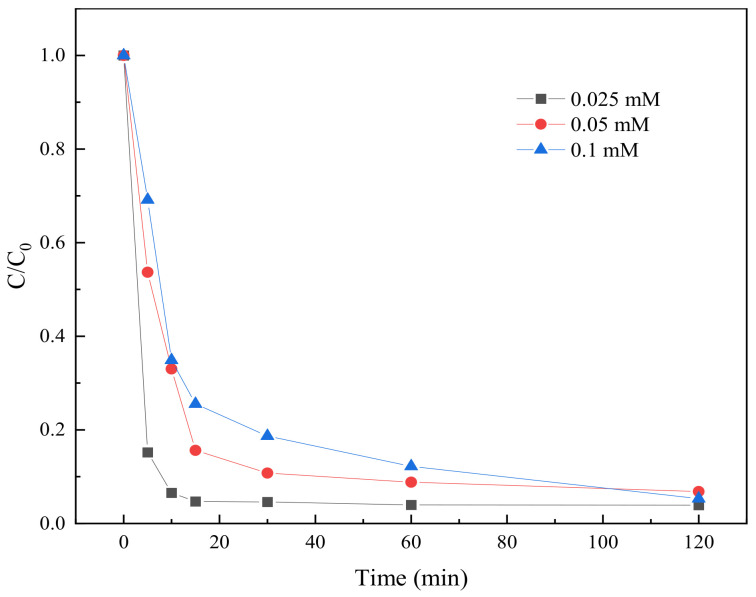
The degradation results of TC at different initial concentrations.

In order to study the effect of initial TC concentration on the degradation effect, three initial concentration gradients were set up in the experiment, namely 0.025 mM, 0.05 mM, and 0.1 mM, respectively. The BC-nZVI material (Fe:C = 1:1) with the best degradation effect in different iron–carbon ratios was selected. The dose of activated material was 10 mg, and the initial TC concentrations were 0.025 mM, 0.05 mM, and 0.1 mM, respectively. The degradation results are shown in Figure 10.

The degradation efficiency of TC was found to decrease with the increase of the initial concentration through three different initial concentration gradients of TC set experimentally. This might be due to the relative reduction of active sites available for TC degradation at higher initial concentrations, leading to a decrease in degradation efficiency, as well as a decrease in the degradation of TC due to competitive reactions between free radicals. In addition, the TC degradation rates under all initial concentration conditions increased significantly with the extension of reaction time, which indicated that the BC-nZVI materials had good persistence and stability.

#### 2.3.5. Effect of PS Concentration on TC Degradation

**Figure 11:**
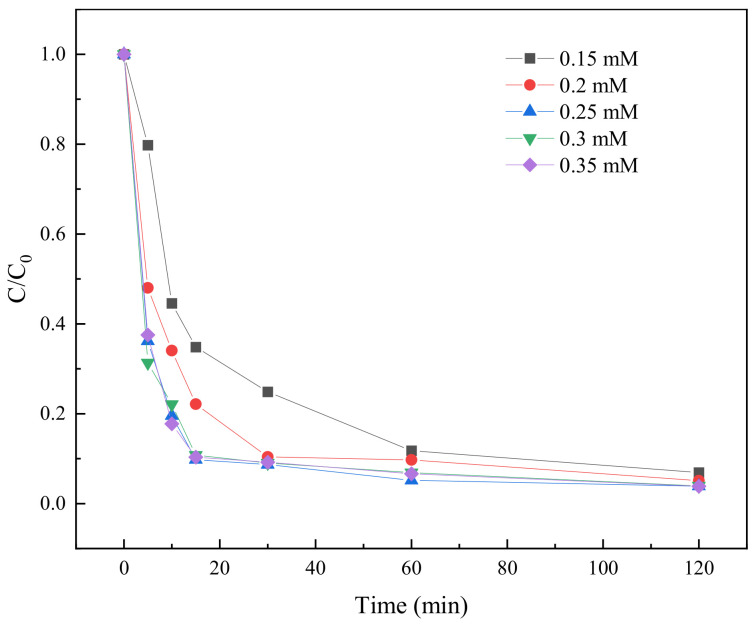
The degradation results of TC at different PS concentrations.

PS concentration is an important factor that affects the degradation effect of TC. In order to investigate the effect of PS concentration on the TC degradation effect, the concentration gradient was set to be 0.15 mM, 0.2 mM, 0.25 mM, 0.3 mM, 0.35 mM, respectively. The BC-nZVI material with a better degradation effect among different iron–carbon ratios was selected (Fe:C = 1:1) and the dose of activated material was 10 mg. The degradation results are shown in Figure 11.

Figure 11 showed that the TC degradation rate increased with increasing the PS concentration. The highest degradation rate of TC was achieved at a PS concentration of 0.25 mM, but when the PS concentration continued to increase to 0.3 mM and 0.35 mM, the degradation rate did not increase significantly. This might be due to the fact that at a certain range of PS concentration, PS can sufficiently react with BC-nZVI to generate enough free radicals to degrade TC [43]. However, when the PS concentration exceeded this range, the generation of free radicals might have reached saturation and therefore the degradation rate no longer increased.

#### 2.3.6. Effect of Different pH Values on TC Degradation

**Figure 12:**
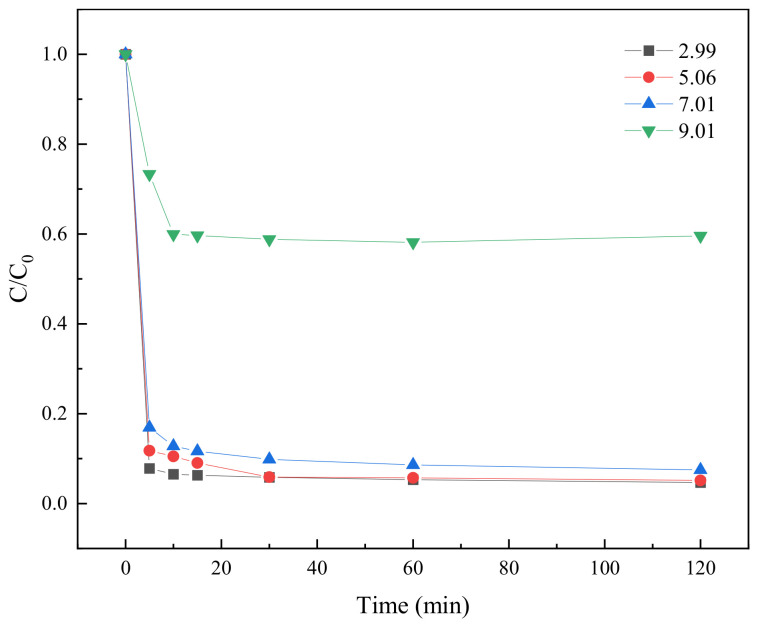
The degradation results of TC at different pH values.

In groundwater, pH is a key parameter in the degradation process of organic contaminants, which significantly affects the generation and activity of free radicals. In this study, the degradation of TC at initial pH values of 3, 5, 7, and 9 was investigated under the conditions of an initial TC concentration of 0.025 mM, a BC-nZVI dosage of 10 mg, and a PS concentration of 0.25 mM. The degradation results of TC at different pH values are shown in Figure 12.

As can be seen from Figure 12, the highest TC degradation rate was observed at pH 3. This may be because the surface charges of nZVI increased in acidic environments, which made it easier for them to come into contact with the TC molecules and promote their degradation. In addition, the activation of PS might be more effective under acidic conditions, and more SO_4_^−^· was generated. These free radicals have strong oxidation and could effectively destroy the molecular structure of TC [44]. However, the degradation efficiency gradually decreased with increasing of pH, especially under alkaline condition at pH 9, where the TC degradation rate was drastically reduced. This might be due to the fact that an oxidized film may form on the surface of nZVI in alkaline environments, hindering its contact with TC [45]. Meanwhile, alkaline conditions may lead to a decrease in the activation efficiency of PS and a decrease in the number of generated radicals, thus affecting the TC degradation efficiency.

#### 2.3.7. Effect of HCO_3_^−^ on TC Degradation

The presence of HCO_3_^−^ in the aqueous environment may have an effect on the degradation of TC by the BC-nZVI/PS system. In this study, six different concentrations of HCO_3_^−^ solutions (0 mM, 0.5 mM, 1 mM, 5 mM, 10 mM, and 25 mM) were used to investigate the effect of HCO_3_^−^ on the degradation of TC.

**Figure 13:**
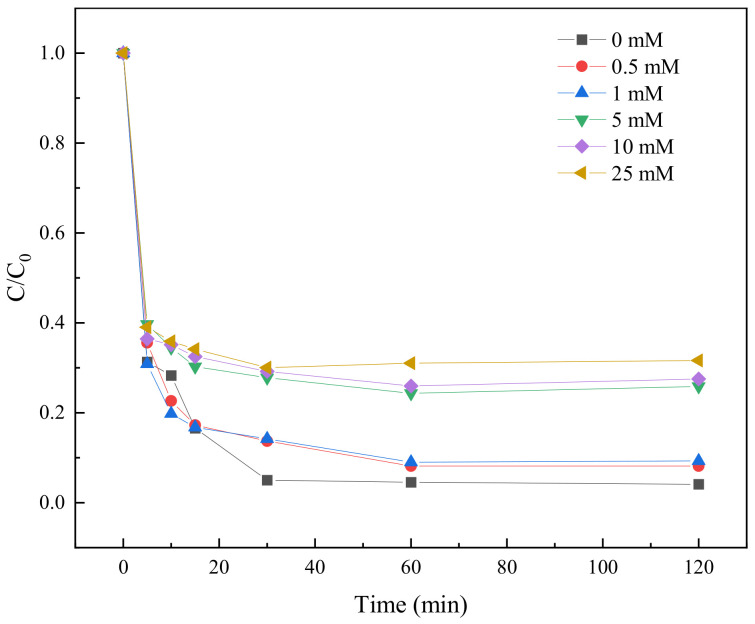
The influence of HCO_3_^−^ on the degradation of TC.

As shown in Figure 13, the presence of HCO_3_^−^ had a significant effect on the degradation efficiency of the BC-nZVI/PS system. In the absence of HCO_3_^−^, the degradation efficiency could reach 71.13% in 5 min, while the degradation rate decreased to 65.42% in 5 min in the presence of 25 mM HCO_3_^−^. The degradation rate gradually increased with the increase of reaction time, and at 120 min, the degradation rate reached 99.34% in the HCO_3_^−^ free condition, while the degradation rate was only 72.81% in the 25 mM HCO_3_^−^ condition. These data indicated that an increase in HCO_3_^−^ concentration inhibited the degradation efficiency of TC. The reason for this analysis might be that HCO_3_^−^ reacted with the free radicals generated by the BC-nZVI/PS system to form more stable carbonate radicals, thus reducing the oxidation of TC. In addition, HCO_3_^−^ might also form stable complexes with Fe^2+^, reducing the effective concentration of Fe^2+^, which in turn affected the activation of PS.

#### 2.3.8. Effect of Cl^−^ on TC Degradation

In order to explore the influence of Cl^−^ in water environment may have on in BC-NZVI/PS system, six different concentrations of Cl^−^ solutions (0 mM, 0.5 mM, 1 mM, 5 mM, 10 mM, and 25 mM) were used to investigate the influence of Cl^−^ on TC degradation.

**Figure 14:**
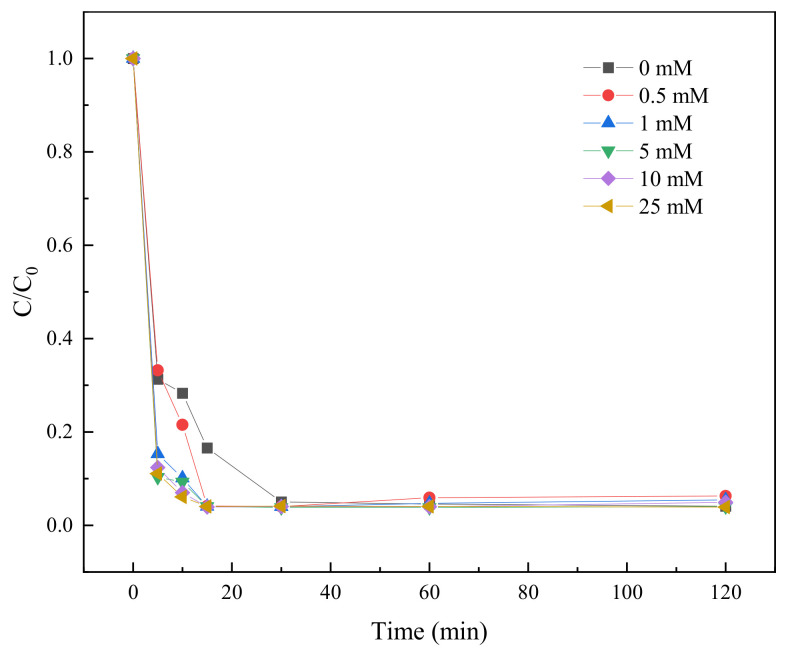
The influence of Cl^−^ on the degradation of TC.

As shown in Figure 14, with the increase of Cl^−^ concentration, the degradation rate of TC showed a certain increasing trend at the beginning of the experiment, especially in the range of 1 mM to 25 mM. This might be because that the appropriate amount of Cl^−^ could promote the activity of nZVI and enhanced its reduction on PS, thus improving the TC degradation efficiency. However, after 60 min, the degradation rate of the high-concentration Cl^−^ sample (25 mM) was slightly decreased. This might be due to the competitive adsorption of excess Cl^−^ with the active sites on the surface of nZVI or the generation of more chlorinated by-products, which might inhibit the further degradation of TC. In addition, the degradation rate of all samples reached more than 99% from 15 min onwards, showing that the BC-nZVI/PS system has an extremely high degradation capacity for TC. However, the effect of Cl^−^ concentration on the degradation efficiency showed different trends at different time points, suggesting that the degradation process might be affected by a variety of factors, such as Cl^−^ concentration, reaction time, and possible synergistic or inhibitory effects. Overall, these results suggested that Cl^−^ could promote the degradation of TC within a certain concentration range, but either too high or too low a concentration may negatively affect the degradation efficiency. Therefore, when applying the BC-nZVI/PS system for TC degradation in water bodies, the effect of Cl^−^ concentration should be taken into account to achieve the best degradation effect.

#### 2.3.9. Effect of SO_4_^2−^ on TC Degradation

The presence of SO_4_^2−^ in the aqueous environment may have an effect on the TC degradation by the BC-nZVI/PS system. Therefore, in this study, six different concentrations of SO_4_^2−^ solution (0 mM, 0.5 mM, 1 mM, 5 mM, 10 mM, and 25 mM) were used to investigate the effect of SO_4_^2−^ on the degradation of TC.

**Figure 15:**
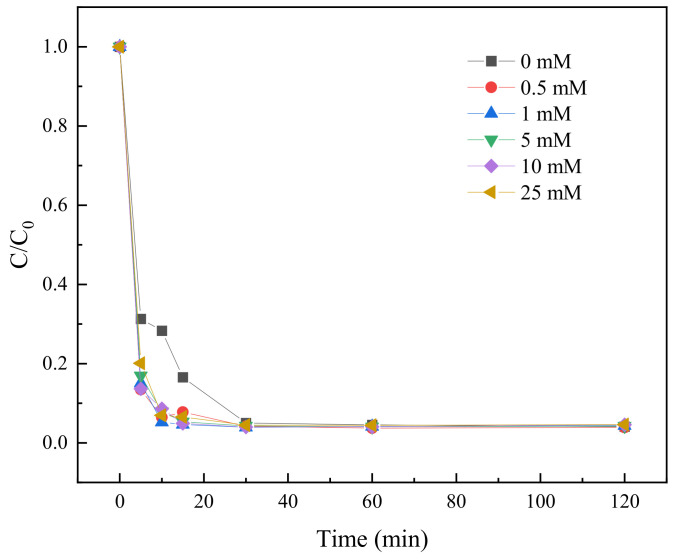
The influence of SO_4_^2−^ on the degradation of TC.

As shown in Figure 15, there was an effect of different concentrations of SO_4_^2−^ on the degradation rate of TC. At the time of 5 min, the degradation rate was 71.13% in the SO_4_^2−^ free condition, while the degradation rate increased to 89.61% at a SO_4_^2−^ concentration of 0.5 mM, which suggested that the low concentration of SO_4_^2−^ may promote the degradation of TC. However, when the SO_4_^2−^ concentration was increased to 25 mM, the degradation rate decreased to 82.76%, which could be attributed to the competitive reaction between the high concentration of SO_4_^2−^ and the actives in the BC-nZVI/PS system, which inhibited the TC degradation.

With the extension of reaction time, the TC degradation rate increased under all conditions, in which the degradation rate reached more than 98% in the time period from 30 min to 120 min, showing that the BC-nZVI/PS system had a good long-term degradation ability on TC. Especially in the range of SO_4_^2−^ concentration from 0.5 mM to 10 mM, the degradation rates at 60 min were all over 99%, which further confirmed that the appropriate amount of SO_4_^2−^ could effectively promote the degradation of TC.

In the BC-nZVI/PS system, moderate amounts of SO_4_^2−^ may facilitate TC degradation by providing additional electron acceptors or participating in the generation of new active species. However, when the concentration of SO_4_^2−^ is too high, the degradation efficiency may be reduced by inhibiting the reduction reaction in the system or consuming too many active species. Therefore, controlling the concentration of SO_4_^2−^ is critical for optimizing the degradation efficiency of the BC-nZVI/PS system in practical applications.

#### 2.3.10. Raw Water Test

**Figure 16:**
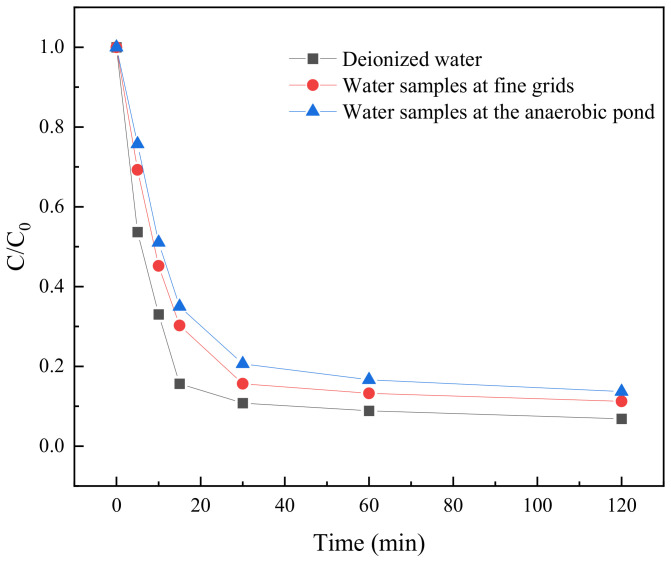
Degradation effect of BC-nZVI/PS system on TC in raw water.

The optimal parameters of BC-nZVI/PS system were obtained, i.e., the dosage of BC-nZVI with 1:1 iron to carbon ratio was 10 mg, the initial concentration of TC was 0.05 mM, the dosage of PS was 0.25 mM, and the pH was 7. This was used as the parameter of the raw water experiments for the degradation of TC on the water samples at the fine grating and at the anaerobic tank to simulate the actual wastewater treatment process. The degradation results were shown in Figure 16.

Figure 16 showed that in the raw water experiment, the degradation rate of TC gradually increased with the increase of reaction time, which indicated that the BC-nZVI/PS system had a good degradation effect on TC under these conditions. In terms of water quality index parameters, the concentrations of COD, ammonia, TP and TN were generally higher in the water samples at the fine grid than that at the anaerobic tank, which could be attributed to more organic matter and nutrients contained in the water samples at the fine grid. However, despite these differences, the BC-nZVI/PS system showed high TC degradation rates in both water samples, particularly above 85% after 60 min of reaction. These data indicated that the BC-nZVI/PS system had potential application in practical water treatment processes and can effectively degrade TC in water.

### 2.4. Mechanism of TC Degradation in Water by BC-nZVI/PS and Analysis of Degradation Products

#### 2.4.1. Free Radical Quenching Experiment

Under the conditions of TC initial concentration of 0.025 mM, BC-nZVI dosage of 10 mg, and PS concentration of 0.25 mM, tertiary butyl alcohol (TBA), methanol (MeOH), p-benzoquinone (PBQ), and histidine (His) were used as the trapping agents, respectively, to study the degradation effect of the BC-nZVI/PS system on TC at different pH values [41]. Among these trapping agents, TBA was specifically used to scavenge the effects from hydroxyl radicals (·OH); MeOH was used as a co-scavenger of ·OH and sulfate radicals (SO_4_^−^·); PBQ was commonly used as a scavenger of superoxide anion radicals (·O_2_^−^); and His was used as a scavenger of singlet oxygen (^1^O_2_). The results are shown in Table 3.

As can be seen from Table 3, the degradation of TC in water samples by the BC-nZVI/PS system was weakened by the addition of TBA compared with the blank control group, which indicated that ·OH played an important role in the degradation process. Meanwhile, under the condition of certain pH value, the addition of MeOH to the system could further reduce the TC degradation rate compared with the addition of TBA, which indicated that SO_4_^−^· could also effectively remove the TC in the water body. In addition, the data showed that the effects of PBQ and His on the TC degradation rate were relatively small, which may be because ·O_2_^−^ and ^1^O_2_ were not the dominant factors in the degradation process of TC. It could be seen that by comparing the effects of different scavengers on the TC degradation rate, it is possible to deduce the dominant free radicals that may be present under different pH conditions. For example, the most significant effects of TBA and MeOH on TC degradation were observed at pH ≤ 7, suggesting that ·OH and SO_4_^−^· were the dominant radicals under acidic conditions.

Besides, the experimental results showed that the TC degradation rate decreased continuously with the increasing pH. At pH = 9, the TC degradation rate decreased significantly from 97.03% to 42.11% even without the addition of radical trapping agent. At higher pH, the higher number of OH- ions can directly act as radical scavenger, which would explain the lower TC degradation at pH 9. Kang et al. suggested that this was due to the increase in electronegativity of certain functional groups in the molecular structure of TC under alkaline conditions. Then, it reduced the attack point of free radicals, resulting in an increase in the stability of TC and a decrease in the degradation efficiency [46]. Chen et al. found that the degradation of TC under alkaline conditions was more influenced by other non-radical pathways [47].

#### 2.4.2. Electron Paramagnetic Resonance (EPR) Experiments

**Figure 17:**
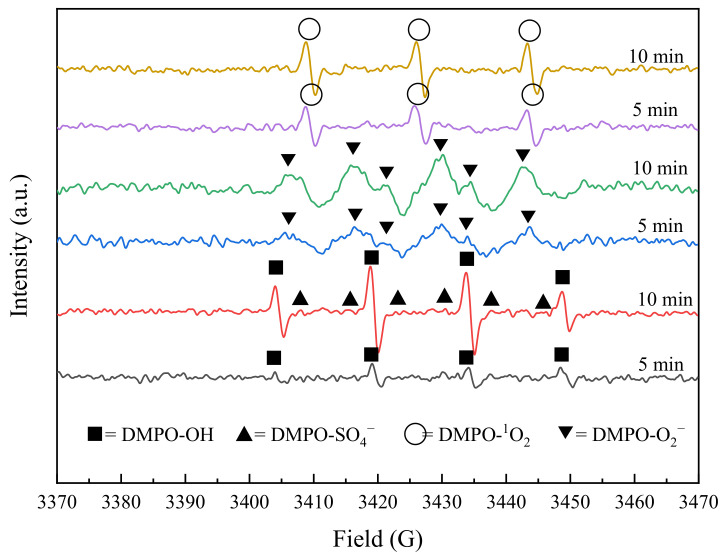
EPR detection spectrum.

Electron paramagnetic resonance (EPR) test was applied to identify and analyze the presence of reactive radicals more precisely. The experiments were carried out at an initial TC concentration of 0.025 mM, BC-nZVI dosage of 10 mg, PS concentration of 0.25 mM, and pH = 7. Meanwhile, 5,5-dimethyl-1-pyrroline-N-oxide (DMPO) was chosen as the capture agent for the experiment, which is due to the fact that DMPO can form stable adducts with a wide range of free radicals and enable the EPR spectra to clearly show the presence and type of free radicals [48]. To delve deeper, the EPR experiments were segmented into three distinct groups based on the radical species being detected. The first group focused on the detection of hydroxyl and sulfate radicals; the second group was tasked with the detection of singlet oxygen; and the third group aimed at detecting superoxide radicals. Each experimental group conducted measurements at two specific time points, 5 min and 10 min, to capture and analyze the transient radicals. This stratified approach not only optimized the experimental workflow, ensuring accuracy and reproducibility of data, but also allowed for a more detailed observation and comparison of different types of radicals. Through this methodology, comprehensive insights into the dynamics of radicals were obtained, which are crucial for understanding their role in environmental chemical processes. The results were shown in Figure 17.

As can be seen from Figure 17, the generation of DMPO-OH was low during the first 5 min of the reaction, from which it can be inferred that the generation of ·OH was not the dominant reaction at this stage. Meanwhile, the DMPO-SO_4_^−^ peak was not detected, which might be due to the insignificant involvement of SO_4_^−^· at this stage or its content was below the detection limit. This was consistent with the previous experimental results that the degradation rate of TC by the BC-nZVI/PS system was low in the first 5 min. As the reaction time was extended to 10 min, the increase in the DMPO-OH peak area indicated a significant increase in the amount of ·OH, which might be related to the enhanced redox activity in the reaction system. The study observed the emergence of the DMPO-SO_4_^−^ peak, although its area was not significantly pronounced. Methanol, as referenced in Table 3, was identified as the most effective scavenger, indicating its potent inhibitory effect on the degradation of tetracycline (TC), which typically suggests a primary role for sulfate radicals in the reaction. However, the near absence of sulfate radical detection by electron paramagnetic resonance (EPR) as depicted in Figure 17 may imply that the high efficiency of methanol in scavenging is not solely through radical quenching. Methanol might also inhibit the degradation of TC by obstructing the adsorption of TC or persulfate (PS) on the BC-nZVI catalyst, thereby impeding the degradation process. Generally, scavengers are capable of not only quenching radicals but also influencing the degradation pathway of TC and PS through interactions with the catalyst [49]. The data showed that the DMPO-^1^O_2_ peak area was small at 5 min and slightly increased at 10 min, which indicated that ^1^O_2_ might start to be gradually generated only in the middle of the reaction. The increase of the DMPO-O_2_^−^ peak area indicated that ·O_2_^−^ was gradually accumulated throughout the reaction and reached a high level at 10 min. Judging from this, the free and non-free radicals activated by the reaction system probably can be expressed by Equations (1)–(5) [50].

BC + S_2_O_8_^2−^ → BC−S_2_O_8_^2−^ (1)

BC−S_2_O_8_^2−^ → BC + SO_4_^−^· (2)

S_2_O_8_^2−^ + 2H_2_O → HO_2_^−^ + 2SO_4_^2−^ + 3H^+^ (3)

S_2_O_8_^2−^ + HO_2_^−^ → SO_4_^–^· + SO_4_^2−^ + O_2_^–^· + H^+^ (4)

HO_2_· → O_2_^–^· + H^+^ (5)

First, S_2_O_8_^2−^ combines with BC via chemical bonding to form a BC−S_2_O_8_^2−^ coordinate. Then, the transfer of electrons within the BC−S_2_O_8_^2−^ coordinate occurs to generate SO_4_^−^· (Equations (1) and (2)). The formation of ·O_2_^−^ radicals can be attributed to the activation of PS by BC (Equations (3)–(5)), and HO_2_^−^ produced during PS activation reacts with S_2_O_8_^2−^ to form ·O_2_^−^ radicals (Equations (3) and (4)). In addition, HO_2_^−^ can also decompose to form ·O_2_^−^ (Equation (5)) [51]. ^1^O_2_ can be generated by the recombination of ·O_2_^−^ and the reaction of ·O_2_^−^ with ·OH (Equations (6)–(14)) [51]. Meanwhile, SO_4_^−^· can react with HO_2_^−^ to form ^1^O_2_ (Equations (3)–(14)).

SO_4_^–^· + OH^−^ → SO_4_^2−^ + ·OH (6)

·OH + ·OH → H_2_O_2_ (7)

·OH + H_2_O_2_ → HO_2_· + H_2_O (8)

O_2_^–^· + ·OH → ^1^O_2_ + OH^−^ (9)

2O_2_^–^· + 2OH^−^ → H_2_O_2_ + ^1^O_2_ (10)

2O_2_^–^· + ·2H_2_O → H_2_O_2_ + 2OH^−^ + ^1^O_2_ (11)

HO_2_· + O_2_^–^· → ^1^O_2_ + HO_2_^−^ (12)

·OH + O_2_^–^· → ^1^O_2_ + OH^−^ (13)

HO_2_· + SO_4_^–^· → HSO_4_^−^ + ^1^O_2_ (14)

#### 2.4.3. Reaction Mechanism Analysis of BC-nZVI/PS System

**Figure 18:**
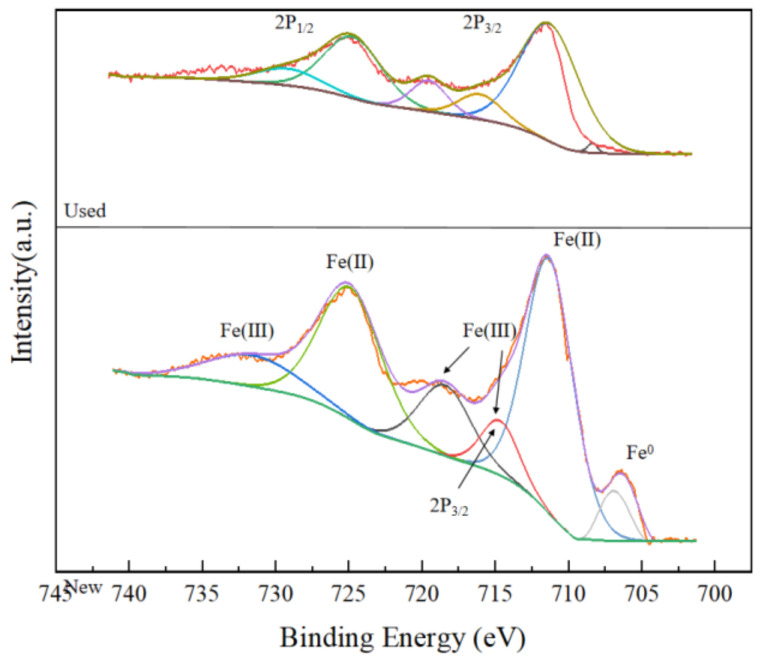
XPS detection spectrum.

In order to further speculate the activation mechanism of the BC-nZVI/PS system, the experiments were carried out using XPS spectroscopy to detect the changes in the chemical state of the Fe element before and after the reaction of the BC-nZVI-activated materials. The results were shown in Figure 18.

As can be seen from Figure 18, Fe(0), Fe(II) and Fe(III) were located at 707.1 eV, 710.2 eV, and 713.2 eV of the Fe_2_p 3/2 spectrum, while they were located at 720.2 eV, 723.3 eV, and 726.3 eV of the Fe_2_p 1/2 spectrum, respectively. Therefore, it can be seen that the characteristic peaks of Fe^0^ disappeared after the reaction compared to the pre-reaction period, while the characteristic peaks of Fe^3+^ and Fe^2+^ changed, indicating to a great extent that iron was involved in the reaction. The reason might be that the introduction of heteroatoms (Fe_3_C) could generate a large number of defects, which in turn could act as catalytic active sites to enhance the nucleophilic addition of PS to generate ^1^O_2_ during PS activation. Furthermore, the active sites on the surface of nZVI (e.g., Fe^0^, Fe^2+^, Fe^3+^, etc.) were probably corroded by PS while participating in the reduction reaction of PS, which led to the nZVI’s activity and stability decrease (Equations (15)–(19)) [52,53].

Fe^0^ → Fe^2+^ + 2e^−^ (15)

Fe^0^ + S_2_O_8_^2−^ → Fe^2+^ + 2SO_4_^2−^ (16)

Fe^0^ + H_2_O + 0.5O_2_ → Fe^2+^ + 2OH^−^ (17)

Fe^0^ + 2H_2_O → Fe^2+^ + H_2_ + 2OH^−^ (18)

S_2_O_8_^2−^ + Fe^2+^ → Fe^3+^ + SO_4_^2−^ + SO_4_^–^· (19)

**Figure 19:**
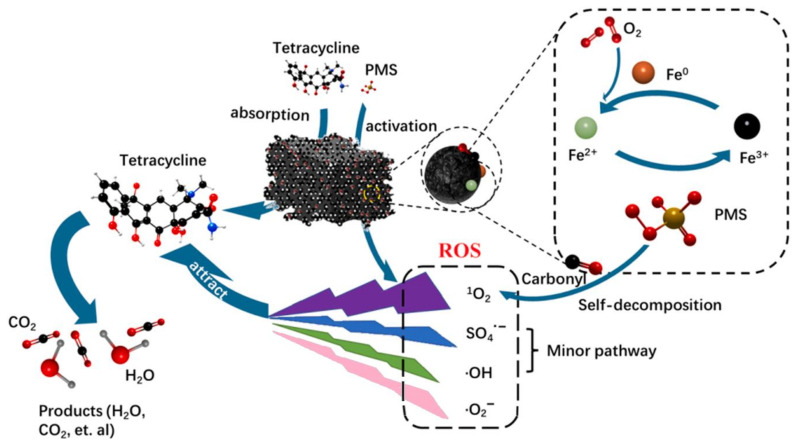
Reaction mechanisms of BC-nZVI/PS system [55].

Based on the above inference, Figure 19 showed the possible mechanisms of TC degradation via radical and non-radical pathways in the BC-nZVI/PS system. First, the mesoporous structure of BC-nZVI increases its specific surface area, which facilitates the adsorptive removal of TC from aqueous solution. In addition, BC-nZVI has excellent activation properties, which are mainly attributed to Fe species and C species. Specifically, Fe species act as a catalyst to effectively promote the chemical reaction, while C species provide a stable carrier to enhance the stability and durability of the material. Thus, the synergistic effect of these two elements enables BC-nZVI to exhibit excellent properties in various chemical reactions, which can efficiently activation of PS to form ·OH, SO_4_^−^·, ·O_2_^−^, and ^1^O_2_ [54]. Ultimately, TC is mineralized to CO_2_, H_2_O, and other small molecules by the combined action of free and non-free radicals.

#### 2.4.4. TC Degradation Pathway Analysis

**Figure 20:**
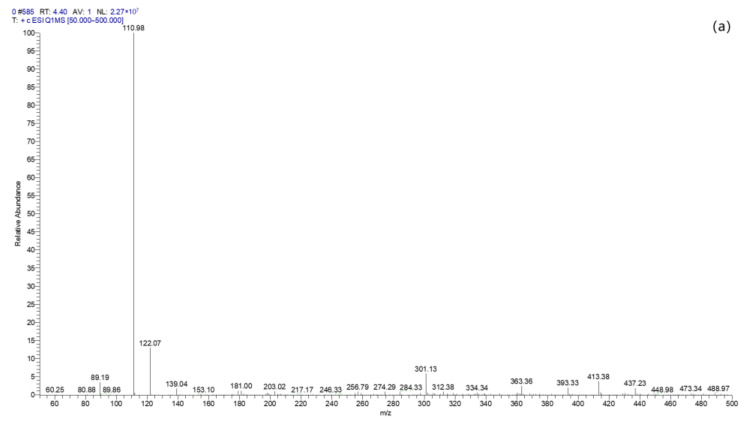

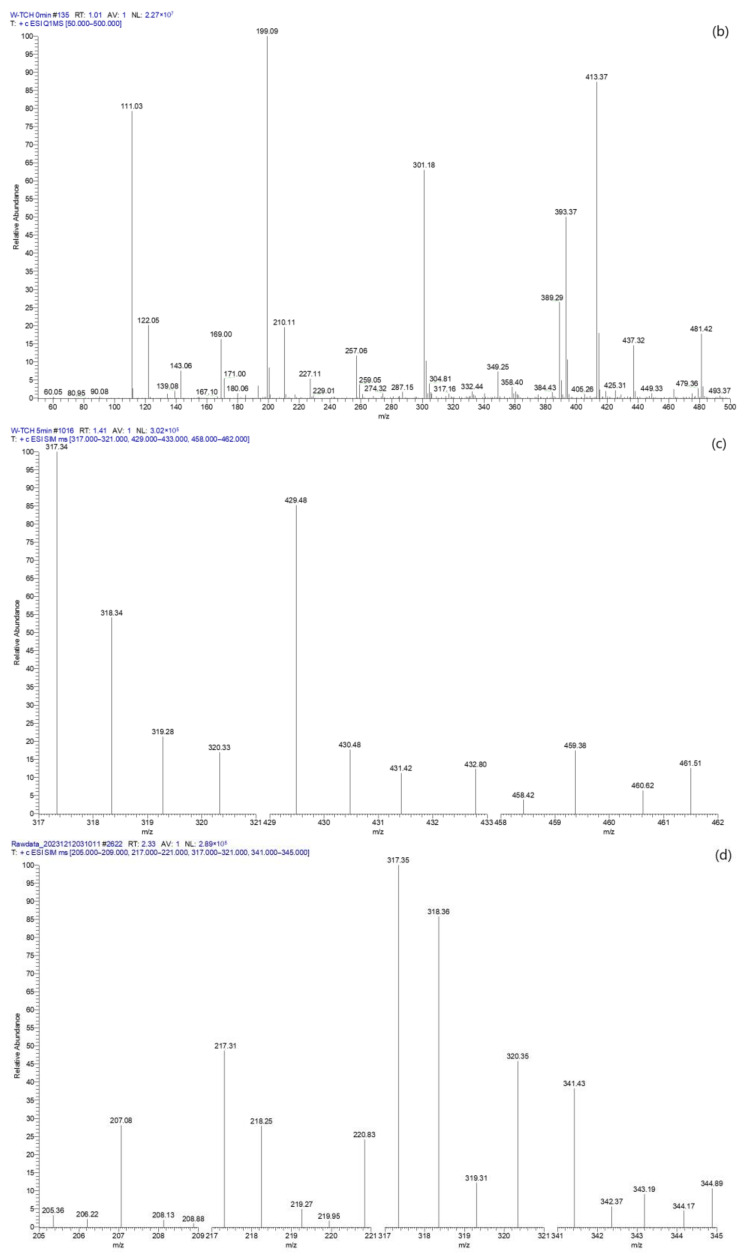

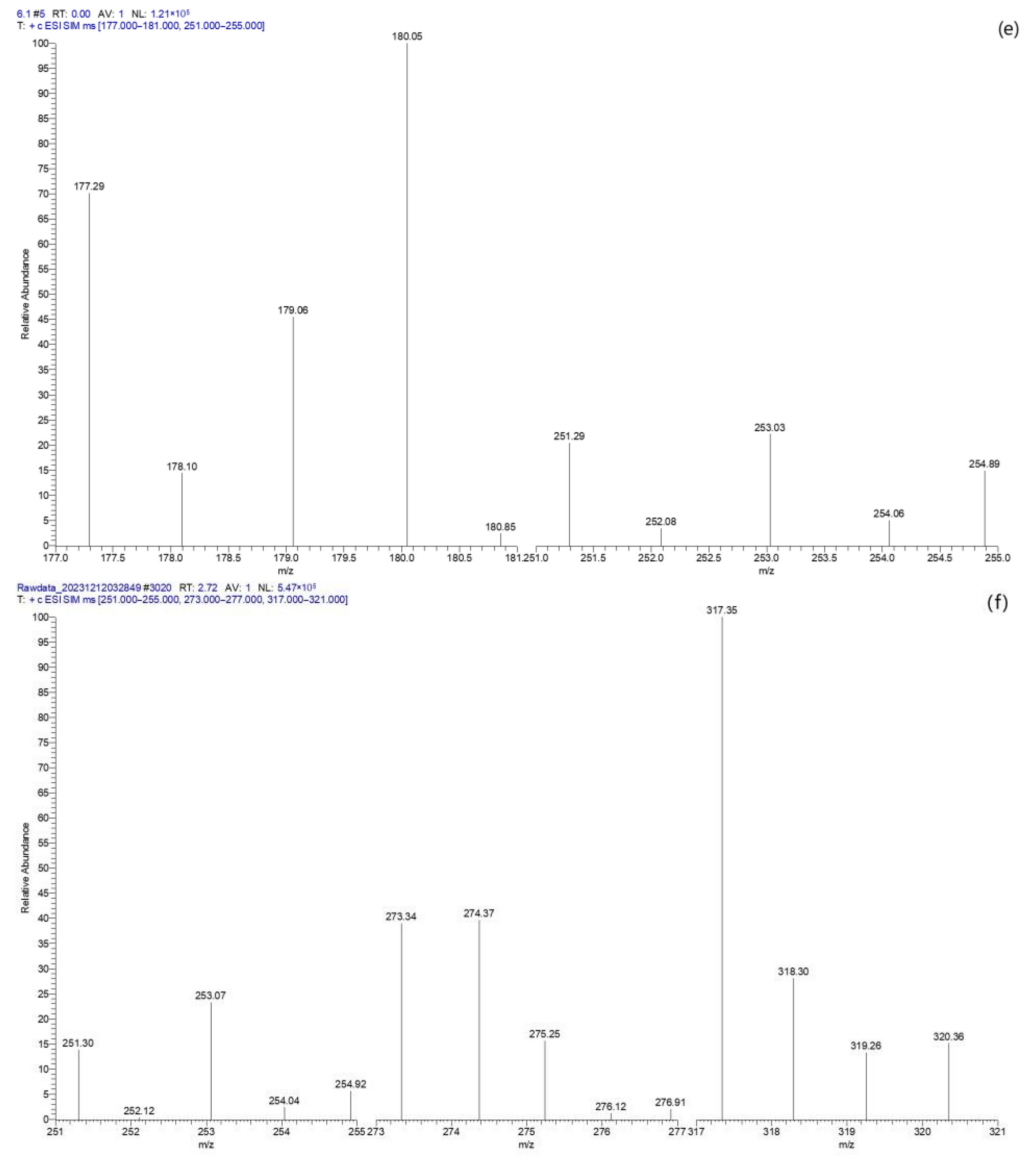
Spectral diagram of TC degradation products. (**a**), The reaction time is 0 min (**b**), The reaction time is 120 min (**c**), The reaction time is 5 min (**d**), The reaction time is 15 min (**e**), The reaction time is 30 min (**f**), The reaction time is 60 min.

In order to analyze the degradation process of TC by BC-nZVI/PS system and its degradation products, after the degradation reaction was carried out for a period of time, the reaction solution was extracted and filtered in time and analyzed using LC–MS detection. Figure 20a showed the LC–MS pattern of the removal of TC by BC-nZVI/PS system at 0 min as a blank experimental group. Figure 20b showed the LC–MS pattern of the removal of TC by BC-nZVI/PS system at 120 min. Then Figure 20c–f showed the LC–MS patterns at each time.

**Figure 21:**
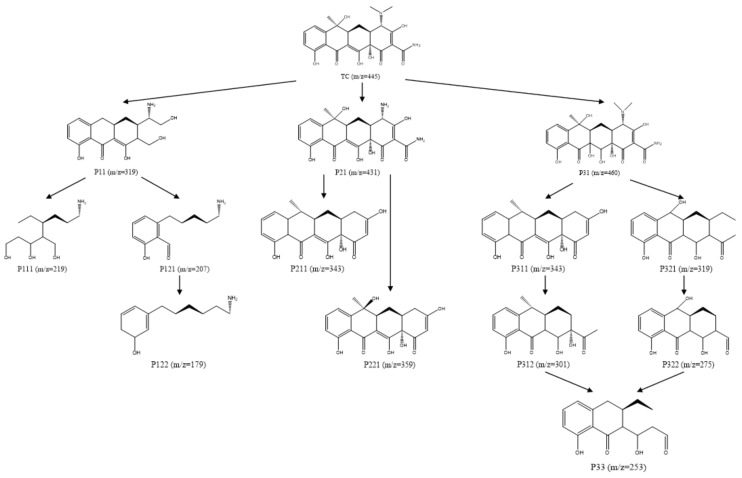
Speculated pathways for TC oxidation degradation.

Based on these molecular structure analyses, three possible pathways for TC degradation were presented in Figure 21. Due to the high electron density of the C=C and C-N bonds in the TC structure, they are more susceptible to attack by free radicals generated during oxidation [56]. In the first degradation pathway, P11 (with a mass–charge ratio of 319) is believed to be an intermediate generated by demethylation, dehydroxylation, and ring-splitting reactions after TC dehydration [57,58]. This intermediate then undergoes a series of reactions to transform into smaller molecular weight intermediates, such as P111, P121, and P122 (mass–charge ratios of 219, 207, and 179, respectively) [59]. In the second pathway, P21 is generated through the N-site dealkylation reaction of TC, while P221 is formed through C-N bond breaking. The two reactions, dehydroxylation and deamidation, occur simultaneously, resulting in the generation of P211 [60]. In the third pathway, P31 is generated through a hydroxylation reaction, and its amide portion is subsequently attacked by reactive oxygen species (ROSs) with concomitant dehydroxylation and deamidation reactions, resulting in the formation of P311 (with a mass–charge ratio of 343) [61]. In a branching pathway, P321 (mass−charge ratio of 319) is formed after ROS attacked the electron-rich conjugated double bond. P311 and P321 further undergo dehydroxylation, ring cleavage, and dealkylation reactions to transform into P312 (mass−charge ratio of 301) and P322 (mass−charge ratio of 275), respectively. Ultimately, these small molecular compounds form the final degradation products through further mineralization [62].

## 3. Materials and Methods

### 3.1. Experimental Material

#### 3.1.1. Experimental Water and Reagents

The experimental water for this experiment is deoxygenated deionized water, and the raw water experimental water is water samples from a sewage treatment plant in Changchun City at the fine grits and anaerobic tank, the current treatment capacity of the plant is 1.0 × 10^5^ m^3^/d, and the water quality parameters are shown in Table 4.

The main reagents used in the experiment are listed in Table 5.

#### 3.1.2. Laboratory Instruments

The main instruments used in the experiment are listed in Table 6.

### 3.2. Experimental Methods

#### 3.2.1. Conventional Water Quality Testing Methods

The test methods for each water quality index in the experiment are shown in Table 7.

#### 3.2.2. Experimental and Detection Methods for PS Activation Patterns

The experiments were designed to investigate the laws and control factors of the BC-nZVI/PS system for degrading TC in water, and to analyze the advantages and mechanisms of the BC-nZVI/PS system by comparing the degradation effects of different classes of activated material reaction systems on TC in water. Furthermore, the effects of different iron to carbon ratio, BC-nZVI dosage, initial TC concentration, PS concentration, initial pH and coexisting anions on TC degradation were investigated to explore the optimal reaction conditions and influence mechanism. This can provide theoretical basis and technical guidance for the engineering application of a BC-nZVI/PS system.

**Figure 22:**
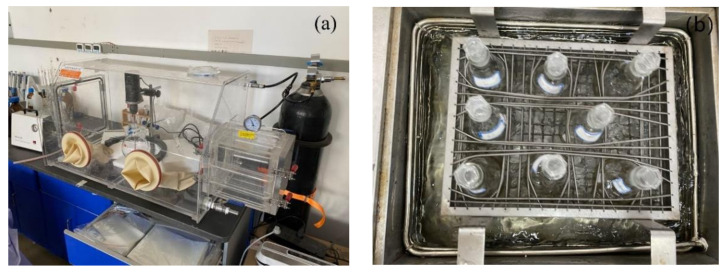
Physical diagram of main experimental equipment. (**a**), Vacuum drying oven (**b**), Water-bath constant tem-perature shaker.

The liquid-phase reduction method was used to prepare the activated material BC-nZVI, and the whole preparation process was carried out in a vacuum glove box, which can effectively avoid the oxidizing effect of oxygen in the air on the material. The device schematic of the vacuum glove box is shown in Figure 22a.

The experiments were conducted to investigate the degradation pattern and influencing factors of BC-nZVI/PS system on TC in water. First, 10 mg of BC-nZVI and 0.25 mM of PS were mixed with 100 mL of TC solution (0.05 mM) in a 250 mL conical flask for batch degradation experiments. The experiments were carried out in a water-bath constant temperature shaker as shown in Figure 22b. A total of 1 mL samples were taken at certain time intervals (0, 5, 15, 30, 60, 90, and 120 min), and the TC concentration was determined by HPLC after dilution with methanol (5 mL) and filtration through 0.22 μm organic-phase filter membranes. In order to examine the reuse performance of BC-nZVI, BC-nZVI was recovered with a magnet after each reaction and washed and dried with deionized water and ethanol before reuse. The kinetic experiments were repeated under the same conditions. All experiments were conducted at pH = 7.0 and two parallel experiments were performed, except for examining the effect of pH on the degradation effect.

#### 3.2.3. Evaluation Method of TC Degradation Effect

Degradation rate is an indicator used to evaluate the degradation effect of organic pollutants in the water treatment process, which is calculated using Formula (20) written as follows:

(20) $$TC\;degradation\;rate\;=\;\frac{C_{t}}{C_{0}}$$

In the formula,

$C_{t}$—TC concentration at reaction time t;

$C_{0}$—initial TC concentration.

### 3.3. Preparation of Materials for Activation Systems

#### 3.3.1. Biochar Modified Treatment

In order to prepare alkali-modified biochar and improve its adsorption performance, the following steps were used in this experiment: first, the dried corn straw biochar powder was passed through a No. 200 mesh sieve to obtain fine-grained biochar. Then, the biochar powder was mixed with 2.0 mol/L NaOH solution at a mass ratio of 1:10 and stirred well to form a slurry. Next, the slurry was placed into a constant temperature water bath, heated to 60 °C, and continuously stirred with an electric stirrer for 2 h to cause a chemical reaction between the biochar and NaOH solution. During the reaction process, the reaction temperature and pH were regularly detected to keep the reaction conditions constant. Afterwards, the reacted slurry was filtered and rinsed with deionized water until neutralized to remove the residual NaOH solution and impurities. During the rinsing process, the deionized water was changed several times until the pH of the rinsing solution was close to 7. Finally, the rinsed solids were dried to constant weight in a vacuum drying oven to obtain alkali-modified biochar. During the drying process, the drying temperature was controlled at 100 °C to avoid structural changes or loss of activity of the biochar.

#### 3.3.2. Preparation of Biochar-Loaded Nano Zero-Valent Iron

**Figure 23:**
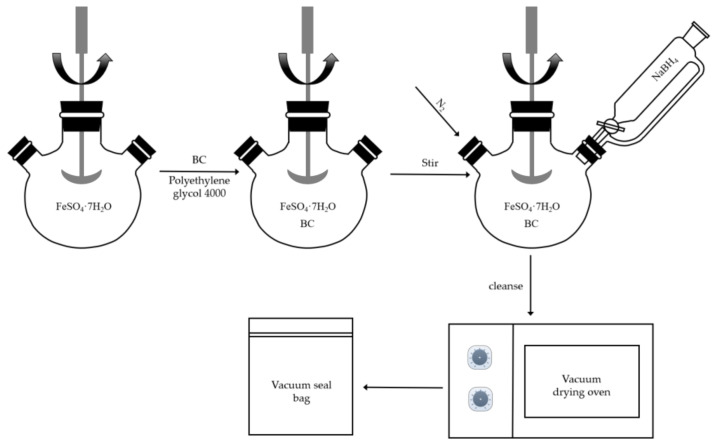
Schematic diagram of preparation of biochar-supported nano zero-valent iron by liquid-phase reduction method.

First, 5 g of FeSO_4_·7H_2_O powder was placed in a 250 mL beaker, 40 mL of deoxygenated deionized water was added and stirred with an electric stirrer (200 r/min) until dissolved. A total of 160 mL of 30% ethanol solution and 0.1 g of polyethylene glycol (4000) were slowly added to the beaker and stirring was continued until well mixed. The ethanol solution and polyethylene glycol (4000) not only regulate the pH of the reaction medium, but also act as dispersants to prevent the aggregation of iron ions. Then, an appropriate amount of BC powder was added to the mixed solution according to different iron–carbon ratios. The iron–carbon ratio is different, and the mass of BC added varies. To prepare BC-nZVI with a 2 g iron–carbon mass ratio of 1:1, the masses of FeSO_4_·7H_2_O and BC used in the experiment are 5 g and 1 g, respectively, as shown in Table 8. After the addition of BC, stirring was continued for 2 h with an electric stirrer (200 r/min) to make full contact between BC and the iron solution. Subsequently, the mixed solution was transferred to a 500 mL three-Necked flask and stirring was maintained under N_2_ atmosphere. Meanwhile, 2.733 g NaBH_4_ was dissolved in 0.1% NaOH solution and placed in a constant pressure dispensing funnel. The NaBH4 solution was added dropwise to the three-necked flask at a rate of 1–2 drops per second to reduce the iron ions to nZVI under N_2_ atmosphere. After the dropwise addition, stirring was continued for 30 min to distribute the nZVI uniformly on the BC. Then, the stirring was stopped and left for 1 h to allow the reaction to proceed fully. Finally, the reaction product was washed alternately with deionized water and anhydrous ethanol 3–5 times under N_2_ atmosphere to remove residual NaBH_4_, NaOH, FeSO_4_, and other impurities. The washed solid material was collected and put into a vacuum drying oven for drying and spare. The preparation process is shown in Figure 23.

#### 3.3.3. Preparation of Zero-Valent Iron Nanoparticles

Preparation of nano zero-valent iron (nZVI) referred to the preparation method of BC-nZVI. The nZVI was prepared by using FeSO_4_·7H_2_O as the iron source, NaBH_4_ as the reducing agent, and ethanol and polyethylene glycol (4000) as the dispersant. The specific steps were as follows: First, 5 g of FeSO_4_·7H_2_O powder was dissolved in 40 mL of deionized water and stirred well with an electric stirrer at 200 r/min. Then, 160 mL of 30% ethanol solution and 0.1 g of polyethylene glycol (4000) were slowly added, and stirring was continued until the mixture was homogeneous. Subsequently, the mixture was transferred to a 500 mL, three-necked flask and the reaction was stirred under N_2_ protection. Meanwhile, 2.733 g NaBH_4_ was dissolved in 0.1% NaOH solution and placed in a constant pressure dispensing funnel. The NaBH_4_ solution was added dropwise into a three-necked flask at a rate of 1–2 drops per second to reduce the ferric ions and to form nZVI under the protection of N_2_. After the dropwise addition, stirring was carried out for another 30 min to distribute the nZVI evenly. Then the stirring was stopped and left for 1 h to allow the reaction to be fully completed. Finally, the reaction product was washed alternately with deionized water and anhydrous ethanol 3–5 times under N_2_ protection until the eluate was neutral. The washed solids were collected and dried in a vacuum drying oven.

## 4. Conclusions

In order to remove TC present in water, BC-nZVI was experimentally prepared as an activator for PS and the effect of BC-nZVI/PS composite system on TC removal was investigated. The microforms, functional group compositions and physical phase changes of BC, nZVI, and BC-nZVI activated materials before and after the reaction were analyzed by various characterization techniques such as SEM-EDS, XRD, BET, XPS, and FT-IR. In addition, the effects of different types of activation materials, different iron-carbon ratios, different reaction conditions (BC-nZVI activation material dosage, initial TC concentration, PS concentration, and pH value) and inorganic anion species on the TC degradation efficiency were experimentally investigated. The reaction mechanism and possible degradation pathways of TC were also systematically revealed by radical type identification and LC–MS analysis, and the following conclusions were obtained:

(1) The experiments were carried out to prepare BC-nZVI-activated materials by liquid-phase reduction method and to characterize and analyze them. SEM-EDS results showed that the alkali-modified BC possessed a porous structure, while nZVI appeared to a certain degree of agglomeration phenomenon, and the BC-nZVI-activated materials demonstrated a good reactivity and structural stability. The successful loading of nZVI was confirmed by XRD analysis, and the XRD analysis confirmed the successful loading of nZVI, and the nZVI and BC mainly existed in the form of physical bonding. BET results showed that the BC-nZVI activated materials had high specific surface area, which was conducive to the enhancement of the adsorption efficiency. XPS and FT-IR analyses revealed the changes in the chemical compositions and functional groups before and after the use of the BC-nZVI activated materials, and the formation of the Fe−O–C bond in the BC-nZVI (new) indicated the successful loading of nZVI, while the formation of the Fe−O–C bond in the BC-nZVI (new) after use indicated the successful loading of the nZVI, whereas the depletion of functional groups and oxidation of nZVI occurred in the used material.

(2) It was found that the BC-nZVI/PS composite system had the best degradation effect on TC, with a degradation rate of 99.34% when the Fe−C ratio was 1:1. In addition, moderate dosage of BC-nZVI activation material could improve the degradation rate of TC, but the effect of excessive dosage was limited, and the optimal dosage was 10 mg. The experimental data showed that the degradation rate of TC was highest when the initial TC concentration was 0.025 mM. The degradation rate of TC in acidic conditions was also higher than that in non-acidic conditions. Meanwhile, the degradation effect of TC under acidic conditions was better than that under alkaline conditions. Common inorganic anions, such as HCO_3_^−^, inhibited the degradation of TC, while moderate amounts of Cl^−^ and SO_4_^2−^ promoted the degradation. Raw water experiments showed that the BC-nZVI/PS composite system has good degradation ability for TC.

(3) Through the free radical burst experiment, the possible reactive oxygen radicals in the reaction system were identified, including ·OH, SO_4_^−^·, ·O_2_^−^, and ^1^O_2_, which play different roles in the degradation of TC under different pH conditions. Meanwhile, the activities of these radicals were confirmed based on EPR experiments. XPS detection showed that Fe0 disappeared and Fe^3+^ and Fe^2+^ increased after the reaction, which indicated that iron was involved in the reaction, and further revealed the possible mechanism of the BC-nZVI activation materials in the reaction. The degradation products after the reaction were analyzed by LC–MS, and three possible degradation pathways were proposed, including carbon−carbon bonds, carbon-Nitrogen bonds, and hydroxyl group reactions, and finally the TC was mineralized into small molecule compounds, such as CO_2_ and H_2_O.

## Figures and Tables

**Table 1:** BET specific surface area of different materials.

Material Type	Specific Surface Area (m^2^/g)	Average Pore Size (m^2^/g)	Total Hole Capacity (m^2^/g)
BC	36.419	5.7531	0.05356
nZVI	43.928	23.8637	0.27467
BC-nZVI (new)	32.9537	23.4526	0.118673
BC-nZVI (used)	91.3207	12.8578	0.311817

**Table 2:** The oxidation degradation parameters of TC in different activation systems.

No.	Fe:C	Activation Systems	TC Concentration (mM)	PS Concentration (mM)	Dosage of Activation Materials PS Concentration (mg)
1	/	blank group	0.05	0	0
2	/	BC	0.05	0	10
3	/	nZVI	0.05	0	10
4	/	PS	0.05	0.25	0
5	/	BC/PS	0.05	0.25	10
6	/	nZVI/PS	0.05	0.25	10
7	1:1	BC-nZVI/PS	0.05	0.25	10

**Table 3:** The impact of MeOH, TBA, PBQ, and His on the degradation of TC at different pH levels.

pH	TC Degradation Rate (%)				
	Blank Group	TBA	MeOH	PBQ	His
3	97.03	73.76	35.69	88.95	87.48
5	94.22	65.64	32.36	86.87	87.4
7	91.51	45.38	31.87	80.63	79.24
9	42.11	22.33	20.26	39.39	36.69

**Table 4:** Effluent quality of a municipal sewage treatment plant.

Water Quality Indicators	Water Samples at Fine Grids	Water Samples at the Anaerobic Pond
	Concentration/Value	Concentration/Value
COD (mg·L^−1^)	207.08	99.56
NH_4_^+^-N (mg·L^−1^)	46.36	34.93
TP (mg·L^−1^)	5.56	13.66
TN (mg·L^−1^)	38.498	30.697
pH	7.23	7.20

**Table 5:** Experimental reagents.

Reagent	Molecular Formula	Purity	Manufacturer
Tetracycline hydrochloride	C_22_H_24_N_2_O_8_·HCl	98%	Maclean’s (Toronto, ON, Canada)
Ferrous sulfate heptahydrate	FeSO_4_·7H_2_O	AR	Tianjin Xinbute (Tianjin, China)
Sodium Borohydride	NaBH_4_	AR	Tianjin Xinbute
Anhydrous ethanol	CH_3_CH_2_OH	AR	Tianjin Xintong (Tainjin, China)
Polyethylene glycol (4000)	H(OCH_2_CH_2_)nOH	AR	Sinopharm Reagent (Shanghai, China)
Sodium hydroxide	NaOH	AR	Aladdin (Los Angeles, CA, USA)
Methanol solution	CH_3_OH	AR	Thermo Fisher (Waltham, MA, USA)
Formic acid solution (0.1%)	HCOOH	AR	Thermo Fisher (Waltham, MA, USA)
Potassium persulfate	K_2_S_2_O_8_	-	Maclean’s (Toronto, ON, Canada)
Biochar	-	-	-

**Table 6:** Experimental instruments.

Instruments	Instrument Model	Manufacturer
Vacuum Glove Box	AGB-4B	TONGLONG (Changshu, China)
Vacuum drying oven	DZF-6020A	Powerstar Technology (Shenzhen, China)
Electric Stirrer	LC-ES-60SH	Li-Chen Technology (Shanghai, China)
Water Bath Oscillator	SHZ-92A	Shaying Scientific Instrument (Shanghai, China)
Magnetic Stirrer	90-2	Changzhou Jintan Kexing Instrument (Changzhou, China)
Electronic Balance	BSA223S	Jena (Shanghai, China)
Numerical control ultrasonic cleaner	KQ3200DE	Linshang Technology (Shenzhen, China)
Constant temperature water bath	SHHW21.600ALL	Tianjin Guanze (Tianjin, China)
Negative pressure vacuum pump	SCJ-10	Changchun Jida-Swan (Changchun, China)
pH meter	PHS-3E	Leimagnet (Shanghai, China)
Liquid Chromatography–Mass Spectrometry	Thermo Scientific TSQ Fortis Plus	Thermo Fisher (Waltham, MA, USA)
LC–MS	Agilent1260	Agilent (Santa Clara, CA, USA)

**Table 7:** Test methods of water quality indicators.

Indicator	Measurement Methodology
NH_4_^+^-N	Nano Reagent Spectrophotometry
TP	Ammonium Molybdate Spectrophotometry
TN	Alkaline Potassium Persulfate Elimination UV Spectrophotometry
COD	Rapid Elimination Method
pH	Portable pH meter

**Table 8:** Variation of biochar mass with carbon–iron ratio in FeSO_4_ solution.

Iron–Carbon Mass Ratio	BC (g)	FeSO_4_ (g)
2:1	0.5	5
1:1	1	5
1:2	2	5
1:4	4	5
1:6	6	5

## Data Availability

The original contributions presented in the study are included in the article, further inquiries can be directed to the corresponding author/s.
